# Oral Anticoagulant Use Among Residents in VA Long-Term Care With Atrial Fibrillation After the 2014 ACC/AHA Guideline

**DOI:** 10.1016/j.jamda.2026.106439

**Published:** 2026-08-26

**Authors:** Veena Manja, Yongmei Li, Michael A. Steinman, Sei Lee, Laura A. Graham, Bocheng Jing, Kathy Fung, Xiaojuan Liu, Steven M. Asch, Michelle C. Odden

**Affiliations:** a Department of Medicine, VA Northern California Healthcare System, Mather, CA, USA; b Department of Medicine, UC Davis School of Medicine, Sacramento, CA, USA; c Department of Epidemiology and Population Health, Stanford School of Medicine, Stanford, CA, USA; d Division of Geriatrics, San Francisco Veterans Affairs Health Care System, San Francisco, CA, USA; e Department of Medicine, University of California, San Francisco, San Francisco, CA, USA; f Health Economic Resource Center, VA Palo Alto Health Care System, Palo Alto, CA, USA; g Division of Pharmacoepidemiology and Pharmacoeconomics, Department of Medicine, Brigham and Women’s Hospital and Harvard Medical School, Boston, MA, USA; h VA HSR&D Center for Innovation to Implementation, VA Palo Alto Health Care System, Palo Alto, CA, USA; i Division of Primary Care and Population Health, Stanford University School of Medicine, Stanford, CA, USA

**Keywords:** Atrial fibrillation, stroke prophylaxis, oral anticoagulation, aspirin, nursing home, deprescribing

## Abstract

**Objectives:**

In 2014, the American College of Cardiology/American Heart Association moved away from recommending aspirin in atrial fibrillation (AF) and recommended that patients be treated with oral anticoagulants (OACs) to mitigate the risk of stroke. We aimed to evaluate the prevalence and predictors of OACs and/or aspirin use among veterans with AF in Veterans Affairs nursing homes in the era after the guideline publication.

**Design:**

Observational study of veterans admitted from October 1, 2014 to September 30, 2019.

**Setting and Participants:**

Four thousand nine hundred ninety-four nursing home residents with AF and a CHA_2_DS_2_-VASc score of ≥2.

**Methods:**

Multinomial logistic regression was used to compare demographics, comorbidities, functional status, and concurrent antiplatelet medication use in residents who received OAC ± aspirin and aspirin alone, compared with neither.

**Results:**

In the era after the 2014 American College of Cardiology/American Heart Association guideline was released, age-standardized prevalence rates for any OAC (warfarin and direct oral anticoagulant) increased from 31.2% in fiscal year (FY) 2013 to 2014 to 46.6% in FY 2019. In addition, there was a decrease in patients receiving no OAC (21.1%−16.2%) and aspirin (47.7%−39.3%). However, some eligible residents remained untreated. Averaged over FY 2015 to 2019, each decade of ascending age was associated with a lower likelihood of receiving OAC [relative risk ratio (RRR), 0.79; 95% CI, 0.71–0.87], relative to neither anticoagulant nor aspirin use. Black veterans were less likely to receive OAC (RRR, 0.73; 95% CI, 0.58–0.91); Hispanic veterans were less likely to receive OAC (RRR, 0.63; 95% CI, 0.44–0.91) and aspirin (RRR, 0.47; 95% CI, 0.31–0.72). Mild to moderate cognitive impairment, greater disability, and other antiplatelet use were also associated with less OAC use.

**Conclusions and Implications:**

Fewer Veterans Affairs nursing home residents received OACs than recommended by guidelines, with significant differences by age and race/ethnicity. Future research efforts should aim to understand the reasons for low OAC use and to augment the evidence base for anticoagulant use to include frail older adults.

Atrial fibrillation (AF) is the most common sustained cardiac arrhythmia affecting millions of Americans and is strongly associated with thromboembolic stroke.^1^ Stroke risk in AF varies with demographics and chronic conditions and can be mitigated by treatment with oral anticoagulants (OACs); the CHA_2_DS_2_-VASc score is commonly used to predict stroke risk and assist with OAC decisions.^2^ Warfarin was the only OAC option till 2010; the availability of effective, safer, and easier-to-use direct oral anticoagulants (DOACs) is now preferred.^3^ Although a previous American College of Cardiology/American Heart Association (ACC/AHA) guideline for the management of AF allowed aspirin for antithrombotic therapy,^4^ more recent studies, including the Aspirin in Reducing Events in the Elderly trial, fail to show benefit and report an increased risk of bleeding complications with aspirin.^5–7^ Since 2014, the ACC/AHA guideline has moved away from recommending aspirin, and the most recent guideline reaffirms that aspirin is not recommended to reduce stroke risk in patients with AF.^2^

In spite of compelling evidence for effectiveness in older adults,^8,9^ OACs are underutilized. Studies have reported aspirin use instead of anticoagulants in up to a third of patients with AF^10^ and reported a lower probability of OAC prescribing when patients with AF are on aspirin.^11^ While guidelines recommend holding OAC in patients with AF who require percutaneous coronary intervention stenting, the vast majority of patients with AF require continuous OAC treatment. OAC and aspirin have been less studied in nursing home residents, who have a high burden of chronic conditions and an elevated risk of both stroke and bleeding outcomes. A recent cross-sectional study of 4752 nursing home residents with AF found that 39% received OAC and 44% received antiplatelets including aspirin.^12^ But this study did not investigate changes over time and after the 2014 guideline publication.

In this investigation, we evaluated trends in aspirin and OAC use in the post-2014 ACC/AHA guideline era, in patients with AF and a CHA_2_DS_2_-VASc score of ≥2 admitted to a Veterans Affairs (VA) nursing home. Furthermore, we investigated predictors of aspirin and OAC use, compared with use of neither of the medications.

## Methods

Our study included residents admitted to a VA nursing home between October 1, 2014 and September 30, 2019 (prior to the onset of the COVID-19 pandemic) with ≥90 days of stay with a diagnosis of AF based on the International Classification of Diseases (ICD) codes in 1 year before admission and a CHA_2_DS_2_-VASc score of ≥2 (the cohort was almost all male). For the time-trend analysis, we also included residents admitted between October 1, 2012 and September 30, 2014. The Stanford institutional review board approved this study.

Demographics, diagnoses, medications, and functional status were obtained from the VA Corporate Data Warehouse. Chronic conditions were identified using ICD-9/ICD-10 and procedure codes 1 year before and during the nursing home stay. AF was identified by ICD-9 427.3x and ICD-10 I48.91 and I48.92.

Patient-level characteristics included age, sex, race/ethnicity, body mass index (BMI), chronic conditions (hypertension, diabetes, dementia, cerebrovascular disease, coronary artery disease, heart failure, peripheral vascular disease, depression, bleeding disorder, and intracranial hemorrhage). Cognitive Function Scale, activities of daily living, and recent falls in the past 30 days were captured from the Minimum Data Set.

Warfarin, DOACs (dabigatran, apixaban, rivaroxaban, and edoxaban), and aspirin use were determined from Bar Code Medication Administration records. OAC use is defined as use of warfarin or DOACs. We categorized exposure in the first 4 weeks as aspirin alone, OAC ± aspirin, or neither, defined as administration on ≥70% of days, and analyzed prevalence, time trends, and adjusted relative risk ratios. We also assessed other antiplatelets (clopidogrel, prasugrel, and ticagrelor) and selective serotonin reuptake inhibitors because of associated bleeding risks.

We present descriptive statistics to characterize the sample and compare characteristics among the 3 groups (Table 1), using the Kruskal-Wallis test for continuous variables and the χ^2^ test for categorical variables. Age-standardized prevalence was calculated by direct standardization to the fiscal year 13 to fiscal year 14 age distribution. We used multinomial logistic regression to examine factors associated with aspirin alone, OAC ± aspirin, or neither, adjusting for the independent variables above. We used α = 0.05. Analyses were performed in SAS 9.4 (SAS Institute Inc.) and Stata 18 (StataCorp LLC).

## Results

Of the 18,506 long-term care residents admitted during the study duration, 27% (5033) had a diagnosis of AF; of these, 4994 had a CHA_2_DS_2_-VASc score of ≥2. The majority (98%) were male with an average age of 80 years. Baseline demographics and comorbidities are presented in Table 1. A total of 28.7% received aspirin alone, 40.8% OAC ± aspirin, and 30.5% received neither. When using a threshold of ≥50% of days to define use, these numbers were similar: 29.4% received aspirin alone, 44.3% OAC ± aspirin, and 26.4% received neither. Among those on aspirin, 63.3% had a history of coronary artery disease compared with 60.6% on an OAC and 50.8% on neither therapy.

**Fig. 1.**
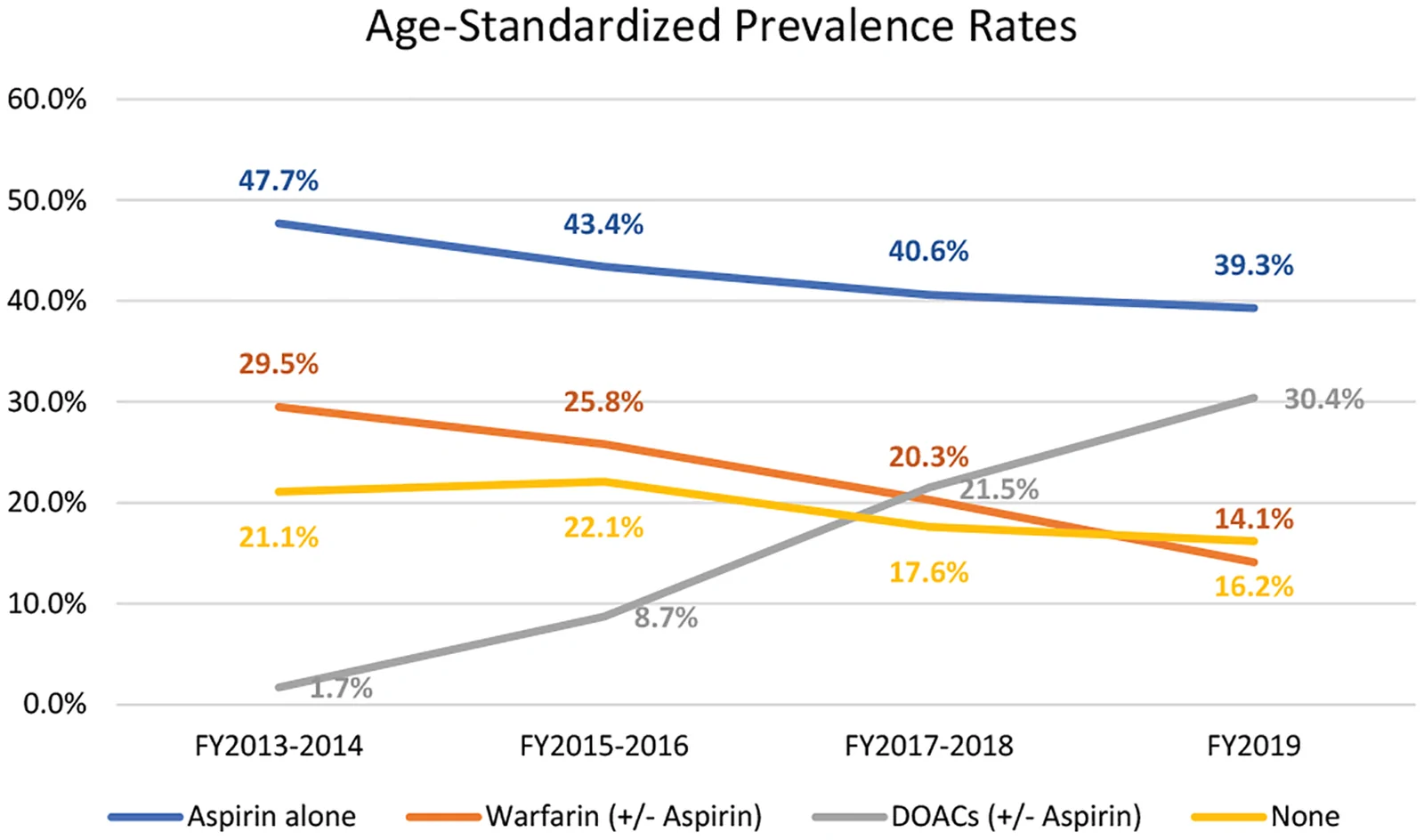
Temporal changes in the use of aspirin alone, warfarin (±aspirin), DOAC (±aspirin), and none in VA nursing home residents with AF and a CHA_2_DS_2_-VASc score of ≥2.

After 2014, age-standardized prevalence rates for any OAC ± aspirin (warfarin and DOAC) increased from 31.2% in 2013 to 2014 to 46.6% in 2019. DOAC use increased over this period, whereas warfarin use declined. In addition, there was a decrease in patients receiving no OAC (21.1%−16.2%) and aspirin (47.7%−39.3%) (Figure 1).

OAC ± aspirin receipt differed significantly based on age, race/ethnicity, and BMI (Table 2). Older age was associated with lower use of OAC (relative risk ratio per 10 years, 0.79; 95% CI, 0.71–0.87). Black residents were less likely to receive OAC, and Hispanic residents were less likely to receive both OAC and aspirin. Residents with a greater BMI were more likely to use OAC.

Other chronic conditions independently associated with greater OAC use included hypertension, diabetes, coronary artery disease, cerebrovascular disease, heart failure, peripheral vascular disease, and depression. Persons with coronary artery disease, cerebrovascular disease, peripheral vascular disease, and dementia were more likely to use aspirin. The presence of a bleeding disorder was associated with lower aspirin use, and a history of intracranial hemorrhage was associated with a lower probability of OAC. Mild or moderate cognitive impairment was associated with lower use of OAC, and greater limitations in activities of daily living were associated with lower use of either medication. Other antiplatelet use was associated with a lower use of OAC.

## Discussion

In the era after the 2014 ACC/AHA guideline was released, anticoagulation use (DOAC or warfarin) increased to nearly 50% in 2019 among residents of VA nursing homes. In parallel, the proportion of those receiving aspirin or no treatment decreased. However, some patients remained untreated with OAC and highlight important differences in the populations that receive guideline-concordant care. OAC use was lower with greater age and among Black and Hispanic individuals and among those with worse cognitive and physical function.

Our results align with previous reports of low OAC use in older adults residing in nursing homes and those with dementia and cognitive impairment.^12,13^ One potential reason for use below guideline recommendations is that studies of OAC in older adults have either excluded or included few with geriatric syndromes, leading to uncertainty in treatment benefits.^14^ This may be of special importance in older nursing home residents and those with worse cognitive and physical function. Functional status, fall risk, and life expectancy are often important considerations for shared decision-making regarding anticoagulant and other medication use in older adults. Another study of 1244 patients (mean age, 75.5 years) with AF reported increased major bleeding episodes and mortality among those on OAC (44% on DOAC) at 24 months of follow-up^15^; this supports our observation of lower use of OAC among residents with a history of intracranial hemorrhage. Furthermore, our study was unable to include factors such as patient preferences, goals of care, and provider evaluation of life expectancy and risks, which would be expected to inform individualized clinical decision-making and could contribute to lower use of OAC. Further data on the effectiveness and safety of OAC among frail older adults and those residing in nursing homes is needed to inform shared decision-making in this population.

Our finding of differences in OAC use among Black and Hispanic older adults is consistent with other research demonstrating racial differences in initiation of OAC after incident AF^16^ and deserves further investigation into the root causes. Factors such as financial strain, medical mistrust, a weak physician-patient relationship, and low health literacy affect racially minoritized populations and can hinder access to guideline-concordant care.^17,18^ Racially minoritized populations also carry a disproportionate burden of chronic conditions, as a result of unequal social conditions, that could influence prescribing decisions. A deeper investigation into these structural and health care factors could identify opportunities to support equitable care.

Strengths of our study include the inclusion of veterans residing in nursing homes across the United States and the availability of the Bar Code Medication Administration data, which represents medications administered, including over-the-counter aspirin use. The primary limitation is that our study population is a selected population and predominantly male; thus, these results may not apply to other nursing home residents. Our study should be replicated in a non-VA nursing home setting to determine whether similar results will be found in both VA and non-VA settings.

## Conclusions and Implications

In summary, OAC use increased substantially in the era after the ACC/AHA guideline was released. We observed significant differences by age, race/ethnicity, and functional status. A better understanding of the benefits of OAC in nursing home residents with AF would help optimize care in these patients.

## Figures and Tables

**Table 1:** Baseline Characteristics of Residents Admitted to Community Living Centers in FY2015 to FY2019, by Medication Categories (n = 4994)

Characteristic	Residents Only on Aspirin N = 1432 (28.7%)	Residents on OAC (±Aspirin) N = 2037 (40.8%)	Residents on Neither Aspirin nor OAC N = 1525 (30.5%)	*P* Value
Age (y)	79.7 (9.3)	76.8 (8.3)	79.9 (9.2)	<.001
Sex				
Male	1407 (98.3%)	2009 (98.6%)	1487 (97.5%)	.046
Race				.017
White	1131 (79.0%)	1629 (80.0%)	1159 (76.0%)	
Black	187 (13.1%)	266 (13.1%)	240 (15.7%)	
Asian or Pacific Islander	19 (1.3%)	19 (0.9%)	22 (1.4%)	
American Indian/Alaska Native	4 (0.3%)	16 (0.8%)	9 (0.6%)	
Multiraces	5 (0.4%)	18 (0.9%)	10 (0.7%)	
Missing value	86 (6.0%)	89 (4.4%)	85 (5.6%)	
Ethnicity—Hispanic	41 (2.9%)	64 (3.1%)	87 (5.7%)	<.001
Weight (lbs)	198.5 (50.9)	221.0 (60.5)	194.6 (49.4)	<.001
BMI	29.0 (7.1)	31.8 (8.3)	28.7 (8.2)	<.001
Hypertension	1323 (92.4%)	1920 (94.3%)	1363 (89.4%)	<.001
Diabetes mellitus	781 (54.5%)	1292 (63.4%)	752 (49.3%)	<.001
Coronary artery disease	907 (63.3%)	1234 (60.6%)	774 (50.8%)	<.001
Cerebrovascular disease	516 (36.0%)	752 (36.9%)	466 (30.6%)	<.001
Heart failure	881 (61.5%)	1372 (67.4%)	878 (57.6%)	<.001
Peripheral vascular disease	439 (30.7%)	565 (27.7%)	322 (21.1%)	<.001
Coagulation/bleeding disorders at admission	28 (2.0%)	78 (3.8%)	46 (3.0%)	.007
Intracranial hemorrhage	57 (4.0%)	47 (2.3%)	69 (4.5%)	.001
Depression	666 (46.5%)	900 (44.2%)	661 (43.3%)	.199
Epilepsy	121 (8.5%)	155 (7.6%)	156 (10.2%)	.021
Dementia	738 (51.5%)	803 (39.4%)	730 (47.9%)	<.001
Cognitive Function Scale				<.001
Cognitively intact	653 (45.6%)	1100 (54.0%)	613 (40.2%)	
Mildly impaired	268 (18.7%)	370 (18.2%)	290 (19.0%)	
Moderately impaired	219 (15.3%)	194 (9.5%)	225 (14.8%)	
Severely impaired	26 (1.8%)	35 (1.7%)	31 (2.0%)	
Missing	266 (18.6%)	338 (16.6%)	366 (24.0%)	
Activity of daily living	14.6 (6.8)	14.3 (6.7)	15.9 (7.1)	<.001
Falls in the past 30 d	481 (33.6%)	653 (32.1%)	470 (30.8%)	.501
Selective serotonin reuptake inhibitor use	464 (32.4%)	636 (31.2%)	453 (29.7%)	.282
Other antiplatelet agents (including clopidogrel, prasugrel, and ticagrelor)	361 (25.2%)	411 (20.2%)	303 (19.9%)	<.001

Report mean (SD) for continuous variables and n (%) for categorical variables; *P* values are based on the Kruskal-Wallis test for continuous variables and the χ^2^ test for categorical variables.

**Table 2:** Multinomial Logistic Regression of Participant Characteristics Associated With Aspirin or OAC (±Aspirin) Use

Characteristic	Aspirin vs Neither		OACs (±Aspirin) vs Neither	
	RRR (95% CI)	*P* Value	RRR (95% CI)	*P* Value
Age per 10 y	1.04 (0.94–1.15)	.448	0.79 (0.71–0.87)	<.001
Female vs male	0.74 (0.42–1.29)	.283	0.57 (0.33–0.98)	.041
Race				
White	Reference		Reference	
Black	0.80 (0.63–1.02)	.071	0.73 (0.58–0.91)	.005
Asian/Pacific Islander	0.81 (0.41–1.58)	.535	0.62 (0.32–1.21)	.161
American Indian/Alaska Native	0.48 (0.14–1.67)	.245	0.60 (0.21–1.68)	.329
Multiraces	0.52 (0.17–1.60)	.254	1.12 (0.48–2.65)	.791
Missing	1.15 (0.81–1.63)	.425	0.85 (0.60–1.20)	.357
Ethnicity—Hispanic vs non-Hispanic	0.47 (0.31–0.72)	.001	0.63 (0.44–0.91)	.013
BMI	1.00 (0.99–1.01)	.486	1.03 (1.02–1.04)	<.001
Hypertension	1.25 (0.93–1.69)	.137	1.46 (1.09–1.95)	.011
Diabetes	1.10 (0.92–1.31)	.292	1.30 (1.10–1.53)	.002
Coronary artery disease	1.44 (1.20–1.72)	<.001	1.31 (1.11–1.55)	.001
Cerebrovascular disease	1.22 (1.02–1.46)	.031	1.57 (1.33–1.86)	<.001
Heart failure	0.99 (0.83–1.18)	.913	1.22 (1.04–1.44)	.017
Peripheral vascular disease	1.49 (1.23–1.81)	<.001	1.20 (1.00–1.44)	.049
Bleeding disorder	0.54 (0.31–0.92)	.023	1.21 (0.80–1.84)	.365
Intracranial hemorrhage	0.89 (0.59–1.34)	.563	0.46 (0.30–0.72)	.001
Depression	1.06 (0.88–1.26)	.560	0.84 (0.71–0.99)	.038
Epilepsy	0.80 (0.59–1.07)	.130	0.77 (0.59–1.01)	.059
Dementia	1.35 (1.12–1.63)	.001	1.06 (0.89–1.26)	.511
Cognitive Function Scale				
Cognitively intact	Reference		Reference	
Mildly impaired	0.80 (0.64–1.00)	.045	0.82 (0.66–1.00)	.053
Moderately impaired	0.96 (0.74–1.25)	.755	0.70 (0.54–0.91)	.008
Severely impaired	1.09 (0.60–1.99)	.771	0.91 (0.51–1.64)	.762
Missing value	0.58 (0.46–0.75)	<.001	0.52 (0.41–0.65)	<.001
Activity of daily living	0.98 (0.97–0.99)	<.001	0.98 (0.97–0.99)	<.001
Falls in 30 d prior	1.16 (0.98–1.38)	.083	1.12 (0.95–1.31)	.180
Selective serotonin reuptake inhibitor use	1.11 (0.91–1.35)	.310	1.09 (0.91–1.31)	.362
Antiplatelet use	1.11 (0.90–1.36)	.338	0.73 (0.60–0.89)	.002
